# 571. Effects of alcohol use on tenofovir alafenamide metabolites among persons with HIV: Implications from a pharmacokinetic model

**DOI:** 10.1093/ofid/ofaf695.180

**Published:** 2026-01-11

**Authors:** Sean AvedissianAli Dunbar, Robert B Parker, Steven Laizure, Ryan Coyle, Mary Morrow, Samantha MaWhinney, Lane Bushman, Lucas Ellison, Jia-Hua Zheng, Subhi Al-Zuabi, Kristina Brooks, Ukamaka O Modebelu, Jose Castillo-Mancilla, Peter Anderson

**Affiliations:** University of Colorado, Denver, Colorado; UTHSC College of Pharmacy, Memphis, Tennessee; University of Tennessee, Memphis, Tennessee; University of Colorado, Denver, Colorado; University of Colorado, Denver, Colorado; Colorado School of Public Health, Denver, Colorado; University of Colorado Anschutz Medical Campus, Aurora, Colorado; University of Colorado - Anschutz Medical Campus, Aurora, Colorado; University of Colorado, Denver, Colorado; University of Colorado Anschutz Medical Campus, Aurora, Colorado; University of Colorado Anschutz Medical Campus, Aurora, Colorado; UNMC, Omaha, NE; University of Colorado-AMC; Skaggs School of Pharmacy and Pharmaceutical Sciences / University of Colorado Anschutz Medical Campus, Aurora, Colorado

## Abstract

**Background:**

Tenofovir alafenamide (TAF) is used to treat hepatitis B virus and HIV. TAF undergoes intracellular hydrolysis by carboxylesterase 1 (CES1) in the liver and cathepsin A in peripheral blood mononuclear cells (PBMCs), followed by phosphorylation to the active moiety, tenofovir-diphosphate (TFV-DP). Evidence in hepatocytes suggests ethanol inhibits TAF hydrolysis to TFV via CES1 inhibition, reducing active TFV-DP in the liver.

**Figure:**
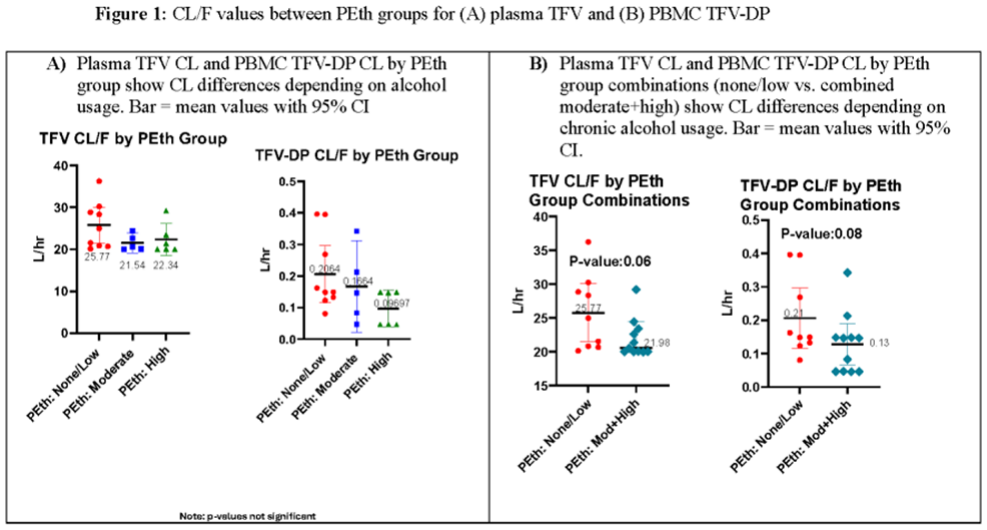

**Methods:**

QUANTI-TAF (NCT04065347) was an observational pharmacokinetic (PK) study where PWH received TAF/emtricitabine (FTC)-based antiretroviral therapy. We evaluated a subset of 20 PWH who self-reported high (n=10) or no (n=10) alcohol use. Plasma TFV, intracellular TFV-DP concentrations in PBMCs, and phosphatidylethanol (PEth [biomarker for alcohol use]) in dried blood spots (DBS) were quantified using validated LC-MS/MS at 0, 4, 8, and 12 weeks. An oral absorption 2-compartment PK model was used to evaluate if PEth category (< 50 ng/mL, low [n=9]; 50-200 ng/mL, moderate [n=5]; and ≥200 ng/mL, high [n=6]) was associated with apparent clearance (CL/bioavailability [F]) of plasma TFV and PBMC TFV-DP.

**Results:**

A total of 20 virally suppressed PWH were evaluated. Median (IQR) DBS PEth concentrations were 131 ng/mL (62-309). Mean (SD) plasma TFV CL/F generally decreased as PEth category increased, although non-significantly (low: 25.77 L/h [±5.55]; moderate: 25.54 L/h [±1.95]; high: 22.34 L/h [±3.63]; ANOVA p=0.18), Figure 1A. In PBMCs, TFV-DP CL/F also generally decreased as PEth category increased (low: 0.2064 L/h [±0.12]; moderate: 0.1664 L/h [±1.12]; high: 0.09697 L/h [±0.06]; ANOVA p=0.16), Figure 1A. When moderate and high PEth categories were combined, there was also a difference in CL/F values for plasma TFV (p=0.06) and PBMC TFV-DP (p=0.08), Figure 1B.

**Conclusion:**

Alcohol use was associated with lower TFV CL/F in plasma, and TFV-DP CL/F in PBMCs, potentially driven by inhibition of CES1-mediated TAF hydrolysis to TFV in hepatocytes. We believe these observed changes in CL/F are likely driven by changes to F. We hypothesize that increased TFV-DP in PBMCs represents a shunting of TAF delivery from hepatocytes (CES1) to PBMCs. Alcohol use could contribute to metabolic differences in TFV conversion from TAF, which could have implications for treatment.

**Disclosures:**

All Authors: No reported disclosures

